# Supplier concentration and corporate innovation input

**DOI:** 10.3389/fpsyg.2022.879706

**Published:** 2022-10-24

**Authors:** Meifeng Zou, Xindong Zhang

**Affiliations:** ^1^Economic and Management College,Taiyuan University of Science and Technology, Taiyuan, China; ^2^Economic and Management College, Shanxi University, Taiyuan, China

**Keywords:** supplier concentration, corporate innovation, risk-taking, resource occupied, bargaining power

## Abstract

This paper studies the relationship between supplier concentration and corporate innovation input. The results show that a firm with a higher supplier concentration tends to have lower R&D investments and invention patents. Considering endogenous problems, this negative relationship is still robust by using the instrumental variable regression and propensity score matching method. Mechanism analysis shows that a firm with higher supplier concentration is impaired risk-taking capacity and occupied resources by the big suppliers. Our evidence shows that a deeper exposure to a small set of large supplier bears negative consequences for the firm. This paper sheds new light on the dark side of a high level of supplier concentration on corporate innovation.

## Introduction

A large literature has explored the factors of innovation. However, it is unclear how a firm’s supplier relationship affects its innovation, especially over-relying on several major suppliers, which is defined as supplier concentration in this study. It is a common phenomenon for Chinese manufacturers to rely on major suppliers. On average, one-third of their purchase buy from a small set of big suppliers in our samples. Intuitively, a concentrated supplier base is often cited as a good signal, as it is believed to keep a stable supply and save purchase costs for firms.

However, it may be not a positive factor for China Securities Regulatory Commission (CSRC). They are concerned that a firm over depends on major suppliers induces risk for the firm (Chu, 2012). So, listed firms are required disclosing top five suppliers’ information such as the purchase amount and proportion in the annual report by the CSRC. This requirement provides a good laboratory for the research. The paper focuses on the link between a firm’s supplier concentration and innovation. The prior literature loses sight of examining how a firm with higher supplier concentration affects its financial decisions. The paper is new in revealing significant costs associated with firms’ reliance on large suppliers.

The baseline test reveals a negative association between innovation and supplier concentration. The estimates may be subject to empirical biases. In particular, one may argue that unobserved factors might cause a negative relation between supplier concentration innovation. To deal with the endogeneity issue, the paper adopts the Propensity Score Matching Methods (PSM) and the instrumental variables approach. It is still a negative link after considering these endogenous. Overall, the above tests suggest that the negative correlation between innovation and supplier concentration is not simply driven by self-selection.

The research attempt to identify the underlying economic mechanism through which this link occurs. The results could be explained by three hypotheses. Firstly, the results could be driven by the reasoning that a firm with a higher supplier concentration may face a higher risk, so the firm is forced to reduce R&D expenditure of its high uncertainty. Secondly, the results could be consistent with the views that increased dependence on the major suppliers leads to the firm’s resources being occupied by the suppliers, so the firm cannot afford resources for innovation. Thirdly, the results may be impelled by the reasoning that a firm with high supplier concentration will be weak bargaining power make a transaction with the big suppliers, which further impairs the firm’s profitability, so the firm cannot afford funds for innovation.

The paper contributes to three strands of literature. First, it speaks to a growing literature on the role of the supply-customer relation on corporate decisions. These studies focus on how a firm’s customer concentration affect its financial decision, including capital structure (Banerjee et al., 2008), loan contract terms (Campello and Gao, 2017), financial distress (Lian, 2017), cash holdings (Itzkowitz, 2013), corporate tax avoidance (Huang et al., 2016), and cost of equity capital (Dhaliwal et al., 2016). However, they overlook the other side. Zhang et al. (2020) et al. touch the supplier concentration, but they focus on the cash holdings rather than innovation. Pearson and Trompeter (1994) and Willekens and Achmadi (2003) investigate supplier concentration in the audit services market. They focus on the special industry but overlook other pervasive industries. This paper explores the relation between a firm’s supplier concentration and its innovation. The results show that supplier concentration is indeed associated with lower innovation.

This study is also related to existing work on how supplier-customer relationship affects innovation. Chu et al. (2019) pay attention on a customer’s geographic proximity affect its innovation. Tan et al. (2019) focus on the relation between a firm’s major customer and its innovation. Unlike them, the study focuses on the role of supplier concentration on innovation. To our best knowledge, it is the first paper that focuses on this topic.

Finally, the study is related to hot literature on the determinants of innovation. A rich set of papers explore factors of innovation. These studies investigate factors of innovation including firm characteristics (Kortum and Lerner, 2000; Aggarwal and Hsu, 2014; Chemmanur et al., 2014; Ferreira et al., 2014; Tian and Wang, 2014; Acharya and Xu, 2017), market-wide economic forces (Aghion et al., 2005, 2013; Bloom et al., 2013; Autor et al., 2020) and macro-level social characteristics (Lerner, 2009; Williams, 2013; Bradley et al., 2017; Fang et al., 2017). But most of them pay little attention on the link of supply chain and innovation. We find that a firm’s supplier concentration is a key determinant for its innovation. A firm’s supplier concentration impairs its innovation.

The paper proceeds as follows. “Theoretical analysis and hypotheses development” develops literature and testable hypotheses. “Research design” describes our data and methodology. “Empirical results” reports empirical results. “Mechanisms analysis” discusses the underlying mechanism. “Conclusion” concludes the paper.

## Theoretical analysis and hypotheses development

### Theoretical framework

The theoretical framework in this study follows resource dependence theory (RDT) and the incomplete contracts theory. A big supplier will gain strong bargain power when a firm’s supplier concentration is high. The RDT (Pfeffer and Salancik, 1978) suggests that bargain power imbalance exists between supplier and firm in a resource-dependence relationship, and the firm has less freedom in choosing the most profitable contract terms when its supplier has strong bargaining power. The big supplier with strong pricing power can raise purchase prices and switch cost such as freight, insurance premium, and storage charge, etc., potentially reducing firms’ profitability and thus the resources allocated to R&D investments and innovations.

Furthermore, based on the theory of incomplete contracts, hold-up problems arise when the firm’s transactions are overly concentrated in a few big suppliers (Grossman and Hart, 1986). This theory predicts that when facing supplier concentration and expecting lower profits, the firm will be less willing to engage in innovations and will reduce their R&D investment.

### Hypotheses development

This section proposes the testable hypotheses on supplier concentration and firm innovation. A firm’s supplier concentration affects its innovation may through two mechanisms: risk mechanism and resource occupied mechanism.

Firstly, the risk mechanism. Based on the resource dependence theory (RDT), supplier concentration can be a risk factor for a firm. One of the risk stems from the danger of break-down of suppliers of materials and services (Zhang et al., 2020). On the one hand, a firm with high level of supplier concentration faces significant business risk if any key suppliers stop supplying materials and services. Such as ZTE Corporation and HUAWEI Company, both trapped in a business distress by the prohibited supplying chip by US Commerce Department. On the other hand, a firm with a concentrated supplier base tends to engage in relationship-specific investments. Such investments are generally risky, particularly when the relationship between firms and suppliers becomes unstable. In addition, the relationship-specific investments are more likely to be negative net present value (NPV) projects that are more difficult to abandon and realization.

Another risk stems from high switching cost for firms with high supplier concentration. It is difficult for firms to find alternative suppliers in a short time if the important suppliers get into financial distress, declare bankruptcy or switch to other firms. Consistent with this notion, the existing studies document that a negative influence between supplier concentration and firm’s financial decisions, such as firms hold more cash with precautionary motivation (Zhang et al., 2020).

The last risk derived from invisible contract between firms and suppliers. Based on the theory of incomplete contracts, suppler-customer relationship has costs for the firm. The suppliers do not provide explicit guarantees that they will continue supply materials or services in the future. So, by depending on one or more suppliers for a significant part of the company’s purchase, firms could potentially lose a large portion of provision at once, which could cripple their operating risk.

The consequence of the supply risk is reduction of risk-taking capability for the firm. However, innovation is a high uncertainty and risky activity (Custódio et al., 2019). It needs intensive funds and long-term investment cycle. When facing risk of supplier concentration, the firm cannot afford venture of innovation. Therefore, the RDT predict that firms with high level of supplier concentration will reduce R&D investment and innovations.

Secondly, resource occupied mechanism. On the one hand, the RDT suggests that power imbalance exists between firms and its suppliers. When the firms rely on major suppliers, these suppliers gain strong bargaining power relative to the firms. The suppliers will occupy more resources with the strong pricing power through several methods such as reducing commercial credit term, transfering costs including freight, premium, and storage charge, etc., and requiring payment in advance. The above approaches of suppliers will impair the firms’ profit and occupy their resources. However, corporate innovation needs intensive funds and sustaining resources. When the firms’ resources were occupied by the suppliers and profits were encroached by these suppliers, the firms do not have enough funds to innovate.

On the other hand, supplier-firm relationship might discourage firms spending more on R&D investment and thus adversely affect their innovation outputs. The negative driving force is the big supplier’s strong pricing power when the firms over-rely on these suppliers. Based on the RDT, suppliers have more incentives to bargain when the firms depend on them. When large suppliers bargain over costs and control over the firms’ gross profits, these firms’ profitability will decrease, leaving them with fewer resources to allocate to R&D investments and innovations.

In conclusion, the above argument leads to hypothesis: a firm’s high supplier concentration hinders its R&D investments and innovations.

## Research design

### The sample

The research sample contains all A-share firms listed in the Shanghai Stock Exchange and Shenzhen Stock Exchange. The data is collected from the China Stock Market and Accounting Research Database (CSMAR). The database includes detailed information on suppliers as well as accounting and stock market data for Chinese public firms. The sample period is from 2007 to 2018. This is because Chinese regulator, CSRC, requires listed firms to disclose information of major suppliers such as amount and proportion of purchase since 2007.

The sample is excluded financial firms and firms without supplier and innovation information. The final sample contains 2,206 firms with 8,761 firm-year observations. To mitigate the effect of outliers, all continuous variables are winsorized at 1% level for both tails.

### Variable measures

This research constructs three measures of supplier concentration inspired by the definition of customer concentration proposed by Patatoukas (2011) and Campello and Gao (2017). The first measure of supplier concentration (SC1) is the purchase ratio from largest supplier, the second (SC2) is the purchase ratio from top five suppliers, the third (dum1) is a dummy variable, which equals to one if the purchase ratio is more than 10% from top one suppliers and zero otherwise.

This study evaluates a firm’s innovation along two dimensions: innovation input and output. Based on the prior studies, the measures of innovation input are R&D expenditures divided by total assets (RDI), and logarithm of R&D expenditures (lnRD). The two measures of innovation output are the logarithm of one plus patent counts (allPatent) and one plus invention patent counts (InPatent).

Following the existing literature (Balsmeier et al., 2017), the study takes into account a set of control variables. The control variables include *lev* (total book debt divided by total assets), *tbq* (market value of equity divided by the book value of equity), *size* (natural logarithm of the book value of asset), *cflow* (net cash flow from operating activities divided by net assets), *growth* (Current period revenue minus previous period revenue divided by previous period revenue). *roa* (net profit divided by total assets), *soe* (equals to one if firm’s nature belongs to a state-owned and zero otherwise), *top1* (share proportion of the largest shareholder), age(natural logarithm of the firm age), *zbmjd* (total property, plant, and equipment divided by total assets), and *zzl* (sales divided by total assets). The detailed definition of variables is presented in Table 1.

**Table 1:** The definition of variables.

Variable	Definition
RDI	R&D expenditures divided by total assets
lnRD	logarithm of R&D expenditure
AllPatent	logarithm of one plus patent counts
InPatent	natural logarithm of one plus invention patents counts
dum1	a dummy variable which equals to one if the purchase ratio is more than 10% from top one suppliers and zero otherwise
SC1	purchase ratio from largest supplier
SC2	purchase ratio from top five suppliers
lev	total book debt divided by total assets
tbq	market value of equity divided by the book value of equity
size	natural logarithm of the book value of assets
cflow	net cash flow from operating activities divided by net assets
growth	growth rate of sale revenue
roa	net profit divided by total assets
soe	equals to one if firm’s nature belongs to a state-owned and zero otherwise
top1	the share proportion of the largest shareholder
age	natural logarithm of the firm age
zbmjd	total property, plant, and equipment divided by total assets
zzl	sales divided by total assets

### Summary statistics

Summary statistics of main variables are presented in Table 2. The mean (median) value of *RDI* is 4.34 (3.40) with a maximum of 26.60 and a minimum of 0.01, the standard deviation is 4.5. The mean (median) value of *AllPatent* is 2.25 (2.3) with maximum of 6.45 and the minimum of 0, the standard deviation is 1.73. The mean (median) value of SC1 is 0.15 (0.11) with a maximum of 0.69 and a minimum of 0.02, the standard deviation is 0.13. It suggests that the firm averagely purchases from the largest supplier at least more than 10%. It is a big effect on the firm. The mean (median) value of SC2 is 0.34 (0.30) with a maximum of 0.92 and a minimum of 0.05, the standard deviation is 18.34. That is, on average, a firm purchases 34% from the top five suppliers in our sample. The mean value of dumm1 is 0.67, which suggests that more half of firms depend on major suppliers. All firm characteristics are comparable to those reported in existing studies.

**Table 2:** Summary statistics.

Variable	N	Mean	SD	Min	p50	Max
RD	8,762	4.340	4.500	0.0100	3.400	26.60
InPatent	8,762	1.150	1.280	0	0.690	5.080
AllPatent	8,762	2.250	1.730	0	2.300	6.450
SC1	6,347	0.150	0.130	0.0200	0.110	0.690
SC2	8,762	0.340	0.200	0.0500	0.300	0.920
dum1	8,777	0.670	0.470	0	1	1
lev	8,762	0.420	0.210	0.0500	0.410	0.930
zzl	8,743	23.77	81.36	0.850	4.970	667.9
zbmjd	8,645	4.880	9.180	0.290	2.380	70.43
roa	8,762	0.0400	0.0500	−0.190	0.0300	0.190
size	8,762	21.94	1.180	19.69	21.77	25.62
cflow	8,762	0.0800	0.160	−0.520	0.0700	0.610
growth	8,759	0.180	0.430	−0.510	0.110	2.740
top1	8,762	34.05	14.66	8.120	31.97	72.88
soe	8,762	0.340	0.480	0	0	1
age	8,661	7.600	0	7.590	7.600	7.610

Table 3 presents the correlations among main variables. There is a positive correlation among different proxy variables of firm innovation such as *RDI*, *lnRD*, *InPatent*, and *AllPatent*. The same relationship exists for supplier concentration including SC1, SC2, SC3 and dum1. There is a negative relation between supplier concentration and firm innovation. It shows that firms with high supplier concentration make less R&D investment and less patent counts, as hypothesized earlier.

**Table 3:** Correlation analysis.

Variable	RDI	lnRD	InPatent	AllPatent	SC2	SC1	SC3	dum1
RDI	1							
lnRD	0.242***	1						
InPatent	0.130***	0.384***	1					
AllPatent	0.056***	0.344***	0.797***	1				
SC2	0.032***	−0.231***	−0.152***	−0.177***	1			
SC1	0.0210	−0.145***	−0.129***	−0.161***	0.866***	1		
SC3	0.0190	−0.163***	−0.089***	−0.095***	0.728***	0.895***	1	
dum1	0.0190	−0.089***	−0.104***	−0.139***	0.573***	0.630***	0.288***	1

*, **, *** denote significance at the 10%, 5%, 1% level, respectively.

Furthermore, we compare the firm with big suppliers and without big suppliers, and there is a surprise difference between them. Table 4 presents a difference of mean and median between a firm with major suppliers and without major suppliers. The difference of median in *RDI*, *InPatent*, and *AllPatent* is both economically and statistically significant. A number of other firm characteristics differ between the two groups as well. A Firm with higher supplier concentration tends to be smaller, higher leverage, less R&D investment, and lower asset operating efficiency.

**Table 4:** Comparison of firms with and without major suppliers.

Variables	Median				Mean			
	dum = 0	dum = 1	Difference	*p*-value	dum = 0	dum = 1	Difference	p-value
RDI	3.710	3.520	8.930	0.00^***^	4.670	4.910	−0.240	0.100
InPatent	1.100	0.690	63.17	0.00^***^	1.440	1.160	0.280	0.00^***^
AllPatent	2.890	2.300	117.6	0.00^***^	2.710	2.220	0.490	0.00^***^
lev	0.400	0.380	15.62	0.00^***^	0.410	0.390	0.0200	0.00^***^
zzl	4.200	4.580	9.410	0.00^***^	110.9	34,212	−34,000	0.420
zbmjd	2.470	2.280	12.77	0.00^***^	4.550	6.890	−2.340	0.120
roa	0.0400	0.0300	2.440	0.120	0.0400	0.0400	0	0.200
size	21.91	21.72	42.11	0.00^***^	22.08	21.92	0.160	0.00^***^
cashflow	0.0700	0.0700	1.260	0.260	0.0800	0.0700	0.0100	0.240
growth	0.100	0.110	2.800	0.09^*^	0.390	0.320	0.0700	0.630
top1	31.06	32.35	4.530	0.03^**^	33.37	34.45	−1.080	0.00^***^
soe	0	0	12.26	0.00^***^	0.280	0.320	−0.0400	0.00^***^
age	7.600	7.600	19.93	0.00^***^	7.600	7.600	0	0.03^**^

*, **, *** denote significance at the 10%, 5%, 1% level, respectively.

## Empirical results

In this section, the study first runs the baseline regression and present the baseline results, then address some potential concerns, and conduct some robust tests, leading strong support to the baseline result.

### The regression of supplier concentration on innovation

The study examines the relation between supplier concentration and firm innovation. It constructs panel regression models for baseline tests following Campello and Gao (2017), Dhaliwal et al. (2016), Itzkowitz (2013), etc. The model includes a range of control variables ( $Controls_{i,t}$ ) which influences innovation. $Ind_{j}\;$ are industry dummies that capture industry heterogeneity. Industry classification is based on the 2011 CSRC definitions of the 20 industries. $Year_{t}$ are time dummies that account for intertemporal variation. The model can be written as follows:


(1) $$\begin{array}{c} Innovation_{i,t+1}=\beta_{0}+\beta_{1}SC_{i,t}+\gamma {\mathrm{Controls}}_{i,t}\\ +\beta_{3}{\mathrm{Ind}}_{j}+\beta_{4}{\mathrm{Year}}_{t}+\varepsilon_{i,t}, \end{array}$$

where *i* indicates firms, *t* indicates the year. The dependent variable in this model is $Innovation_{i,t}$ including innovation input and output, measuring by RDI and lnRD based on Tian and Wang (2014), Aghion et al. (2013), Chemmanur et al. (2014), and Luong et al. (2017). The independent variable is $SC_{i,t}$ measuring by dum1, SC1 and SC2.

Table 5 reports the results of regression (1). Columns (1)–(2) report the OLS estimates with the dependent variable *RDI* and *lnRD*. Across the columns (1)–(2) of Table 5, the coefficient on SC2 is negative and both economically and statistically significant. For example, the coefficient of column (2) is –0.5934. The results show that when firms’ procurement is concentrated in one or several big suppliers, the firm will significantly reduce its innovation investment. The results suggest that firms with higher supplier concentration is a negative factor for corporate innovation investment.

**Table 5:** Supplier concentration and corporate innovation.

Variables	Innovation input		Innovation output	
	(1) RDI	(2) lnRD	(3) InPatent	(4) AllPatent
SC2	−0.0076^**^	−0.5934^***^	−0.6712^***^	−0.9927^***^
	(−3.0230)	(−8.2391)	(−9.5912)	(−11.2125)
lev	−2.2444^***^	−0.3105^***^	−0.1976^***^	−0.3028^***^
	(−6.3206)	(−4.0115)	(−2.6459)	(−3.2038)
zzl	0.0000	0.0000	−0.0000	−0.0000
	(0.2125)	(0.5187)	(−1.1502)	(−0.2532)
zbmjd	−0.0030^***^	−0.0001	0.0000	−0.0000
	(−2.8940)	(−0.5103)	(0.1328)	(−0.0469)
roa	1.4240^*^	1.1982^***^	0.6254^***^	0.8979^***^
	(1.8557)	(7.2073)	(3.8748)	(4.3969)
size	−0.3932^***^	0.6882^***^	0.2924^***^	0.3842^***^
	(−5.9559)	(48.0999)	(21.0589)	(21.8706)
cflow	0.0243	−0.0374	−0.0170	−0.0264
	(0.2027)	(−1.4463)	(−0.6738)	(−0.8284)
growth	0.0013	−0.0006	−0.0008	−0.0017^*^
	(0.3801)	(−0.7268)	(−1.1206)	(−1.7801)
top1	−0.0170^***^	0.0020^**^	−0.0017^*^	−0.0012
	(−3.8977)	(2.0793)	(−1.8430)	(−1.0368)
soe	−0.6315^***^	−0.0111	−0.1137^***^	−0.2594^***^
	(−4.1067)	(−0.3324)	(−3.5150)	(−6.3390)
age	60.0351^**^	20.9098^***^	84.0201^***^	131.2856^***^
	(2.2776)	(3.6642)	(15.1543)	(18.7157)
Ind FE	Y	Y	Y	Y
Year FE	Y	Y	Y	Y
Prov FE	Y	Y	Y	Y
Adjust.R^2^	0.2523	0.4637	0.2230	0.2980
F	50.1962^***^	287.3297^***^	82.7561^***^	105.3049^***^
N	8,527	8,490	8,527	8,527

*t*-statistics in parentheses.

* *p* < 0.1;

** *p* < 0.05;

*** *p* < 0.01.

Columns (3)–(4) of Table 5 report the OLS estimates with the dependent variables *InPatent* and *AllPatent*. It shows the same results as columns (1) and (2). The coefficient on SC2 is still negative and both economically and statistically significant across all regressions. It suggests that firms with higher supplier concentration have fewer patent counts. The results indicate that a one-standard-deviation increase in supplier concentration is associated with a 0.99% reduction of patent counts. It is a big effect on the firms.

In a word, the results of Table 5 are consistent with the above hypothesis: a firm’s high supplier concentration hinders its R&D investments and innovations. The evidence show that a more concentrated supplier base is associated with fewer R&D spending and patent counts. It suggests that a firm over depends on its major supplier will hinder its innovation activity.

### Addressing potential endogeneity

There may be endogenous problems between supplier concentration and corporate innovation. On the one hand, some unobservable factors may determine the firm’s supplier concentration and its innovation at the same time; On the other hand, a firm with lower innovation level may more depend on major suppliers.

#### Instrumental variables regressions

To mitigate concerns that being in a relationship and corporate innovation are jointly determined by an unobservable factor, the study uses an instrumental variable. The advantage of instrumental variable is that it can partially solve the problem of missing variable and measurement error. The disadvantages of instrumental variables are as follows: first, because instrumental variables are not unique, the estimators of them are arbitrary; second, because the error term is not observable in fact, it is difficult to find variables that are strictly independent of the error term and highly correlated with the endogenous explanatory variable they replace.

Based on the Dhaliwal et al. (2016), the instrumental variable used in the first stage is the lag of supplier concentration, either SC1 or SC2. First, the study tests the validity of the selected instrumental variables. Specifically, the research performs the under-identification test, weak identification test, and overidentification test with results reported in Table 6. It can be shown that the chosen instrumental variables pass the validity tests. For example, the value of p of LM statistic is close to zero, the Cragg-Donald Wald F statistic is smaller than 5% maximal. It implies that the selected IVs do not suffer from the endogeneity issue. The weak identification and overidentification tests reported in Table 6 also show the adequacy of the choosing IVs.

**Table 6:** Validity test of instrumental variable.

Item	L1.SC1	L1.SC2
**Under-identification test**: Anderson canon. Corr. LM statistic (P-value)	46.283 (0.000)	26.000 (0.000)
**Weak identification test**: Cragg-Donald Wald F statistic	16.519	18.771
Stock–Yogo weak ID test critical values: 5% maximal IV	13.91	13.91
**Overidentification test**: Sargan statistic (P-value)	9.869 (0.5180)	3.400 (0.1817)

Second, Two-Stage Least Squares (2SLS) regressions. With the one-year lagged SC as the IV, Panel A of Table 7 presents the results of the first-stage regression of SC (SC1 and SC2) on IVs (L1.SC1 and L1.SC2). The results show that SC is persistent with the coefficient of 0.662 on SC1 and 0.762 on SC2. Panel B of Table 7 presents the results of the second-stage regressions. Consistent with the findings from the baseline regression, the negative relation between supplier concentration and innovation remains significant at −1.02 and − 0.91 after taking into account the endogenous issue.

**Table 7:** 2SLS regressions.

Variables	SC1	SC2
**Panel A: First-stage of instrumental variables regressions**		
L1.SC1	0.662^***^	
	(23.08)	
L1.SC2		0.762^***^
		(87.31)
**Panel B: Second-stage of instrumental variables regressions**		
	lnRD	lnRD
L1.SC1	−1.0222^***^	
	(−3.723)	
L1.SC2		−0.9110^***^
		(−5.260)
lev	−0.2048	−0.4921^***^
	(−0.944)	(−2.694)
ldbl	−0.0089	−0.0123
	(−0.680)	(−1.061)
zzl	−0.0001	−0.0000
	(−0.294)	(−1.283)
zbmjd	−0.0017^***^	−0.0004
	(−3.033)	(−0.940)
roa	2.1238^***^	1.2647^***^
	(4.917)	(3.644)
size	0.6610^***^	0.6897^***^
	(19.113)	(23.449)
cflow	0.1627	−0.2580^***^
	(0.927)	(−2.871)
growth	−0.0308^***^	−0.0345^***^
	(−2.972)	(−3.404)
top1	0.0061^***^	0.0038^**^
	(3.035)	(2.141)
soe	0.0375	0.0839
	(0.529)	(1.390)
age	18.1639	9.4592
	(1.523)	(0.922)
Ind FE	Y	Y
Year FE	Y	Y
Prov FE	Y	Y
Adjust.R^2^	0.445	0.488
F	1533.76^***^	2258.77^***^
N	1,628	2,120

*t*-statistics in parentheses.

* *p* < 0.1;

** *p* < 0.05;

*** *p* < 0.01.

#### Propensity score matching

In order to mitigate self-selection bias, the study conducts propensity score matching (PSM). The advantage of PSM is that it can reduce the self-selection bias. Its disadvantage is that it requires a relatively large sample size and ignores the imbalance between samples. In the application that follows, first, the research classifies the samples into two groups: (1) the treatment group that contains firms having their SC2 measures greater than the median; (2) the control group that includes firms having their SC2 measures smaller than the median. The PSM technique is based on the likelihood that an observation would be a high supplier concentration firm conditional on observables.

Second, the study uses a logit specification to estimate the probabilities of being a high supplier concentration firm (= 1; 0 = otherwise) on a comprehensive list of observable characteristics, including all control variables. Third, the research uses the predicted probabilities, or propensity scores, from the logit estimate and performs the matching. The research uses neighbor, radius, and kernel matching that allows each treated firm to be matched with multiple controls, running the procedure with replacement.

Table 8 presents the average treatment effect estimates. The research findings are in line with those obtained in the previous panel regressions. For example, the results in neighbor matching and radius matching suggest that firm with higher supplier concentration produce less innovation. There is a significant difference between treat and control group for ATT. It implies that firms relying on big suppliers will reduce corporate innovation. Overall, this suggests that the non-random assignment of high levels supplier concentration to less innovative firms does not explain the findings.

**Table 8:** Average treatment effect for PSM.

Matching method		Treated	Controls	Difference	T-stat
Unmatched		1.55	1.91	−0.359	−10.58
Neighbor matching	ATT	1.55	1.83	−0.283	−7.05
Radius matching	ATT	1.56	1.87	−0.309	−7.45
Kernel matching	ATT	1.55	1.83	−0.277	−7.92

### Robust tests

Even though the study includes as many firm control variables as possible in the empirical models which have been identified in prior research to affect firm innovation, adopts instrumental variables for the endogenous variable, and performs propensity score matching to control self-selection bias, it is not free from endogeneity concerns between supplier concentration and firm innovation. For example, if the supplier concentration measures are capturing nonlinear effects of the control variables on the innovation, then the linear combination of control variables in the analyses might not be sufficient to adequately take it into account the differences between a firm with a concentrated supplier base and those without one.

To estimate nonlinear effects, the study adds quadratic term (that is, SC2_2 and SC1_1) of independent variables (that is, SC2 and SC1) based on the model (1). The estimated coefficients on SC2 and SC2_2 is-0.1726 and 0.4774 for the quadratic model in Table 9. The first column shows that both the linear (SC2) and quadratic terms (sc2_2) are individually and jointly insignificant. The same results are as for the second column. The above results show that there are not nonlinear effects between supplier concentration and firm innovation.

**Table 9:** Robustness tests.

	(1) lnRD	(2) lnRD
SC2	−0.1726	
	(−0.690)	
SC_2	0.4774	
	(0.757)	
SC1		−0.8470^***^
		(−3.057)
SC_1		0.4614
		(1.146)
lev	−0.3038^***^	−0.3698^***^
	(−3.919)	(−3.757)
ldbl	0.0002	−0.0045
	(0.066)	(−1.313)
zzl	0.0000	0.0000
	(0.510)	(0.471)
zbmjd	−0.0001	−0.0020^***^
	(−0.442)	(−5.770)
roa	1.2034^***^	1.7734^***^
	(7.238)	(8.364)
size	0.6909^***^	0.6780^***^
	(48.016)	(39.391)
cflow	−0.0375	0.3626^***^
	(−1.449)	(4.841)
growth	−0.0006	−0.0012
	(−0.744)	(−1.638)
top1	0.0019^**^	0.0028^***^
	(2.044)	(2.704)
soe	−0.0118	0.0318
	(−0.355)	(0.815)
age	20.7160^***^	22.7888^***^
	(3.630)	(3.612)
Ind FE	Y	Y
Year FE	Y	Y
Prov FE	Y	Y
Adjust.R^2^	0.464	0.447
F	265.531^***^	183.208^***^
N	8,490	6,143

Numbers in parentheses are t-statistics, *, **, *** denote significance at the 10%, 5%,1% level, respectively.

## Mechanisms analysis

In this section, the study explores possible economic mechanisms through which a firm’s supplier concentration affects its innovation. The research examines two plausible mechanisms, namely, the risk and resource occupied mechanism.

### Risk mechanism

The findings of a negative link between supplier concentration and corporate innovation suggests that it is risky for firm with the high supplier concentration (Dhaliwal et al., 2016). Based on the section II of “*theoretical analysis*,” supplier concentration risk is related to the firm’s operating activity. The risk forces the firm to be more conservative and reluctant to invest R&D spending. Thus, supplier concentration affects corporate innovation may *via* risk mechanism.

In order to verify whether supplier concentration increases the firm’s risk, in turn, affects innovation, the research measures the risk by Z-score (Altman, 2005). The prior literature shows that the Z-score is negatively correlated with the sustainable operating risk. That is, the sustainable operating risk of firm is higher, the Z-score is lower. So, if the risk mechanism drives the negative relation between supplier concentration and innovation, it should expect to observe significant negative relation between supplier concentration and risk measuring by Z-score. To test the conjecture, the study regresses the supplier concentration measuring for *dum1*, *SC1*, and *SC2* and sustainable operating risk measured by Z-score for emerge market. Specially, Z-score = 3.25 + 6.56×1 + 3.26×2+ 6.72×3 + 1.05×4, where X1 = (current asset-liquid debt)/asset, X2 = retained earnings/asset, X3 = EBIT/asset, X4 = equity/debt), EBIT is short for Earnings Before Interest and Tax.

The results are presented in columns (1)–(3) of Table 10. The coefficient of dum1 on Z-score is –0.021, it is negative and highly significant, suggesting that a firm with higher supplier concentration is more likely to experience high risky, when compared to firms with lower supplier concentration. As shown in columns (2) and (3), the coefficients of SC1 and SC2 are −0.105 and − 0.138, respectively. These results suggest that a firm over depending on major is more likely to increase sustainable operating risk.

**Table 10:** Risk mechanism.

	(1) Z-score	(2) Z-score	(3) Z-score	(4) lnRD	(5) AllPatent
dum1	−0.021^**^				
	(−2.643)				
SC1		−0.105^*^			
		(−1.894)			
SC2			−0.138^***^		
			(−3.564)		
Z-score				0.053^***^	0.019^**^
				(5.394)	(2.342)
lev	−5.918^***^	−6.580^***^	−5.918^***^	−0.335^***^	−0.335^**^
	(−52.683)	(−61.555)	(−52.695)	(−3.157)	(−2.137)
ldbl	0.061^***^	0.053^***^	0.061^***^	−0.012^***^	−0.026^***^
	(16.615)	(16.294)	(16.403)	(−4.056)	(−5.875)
zbmjd	−0.015^***^	0.003^***^	−0.015^***^	0.002^***^	0.000
	(−40.731)	(6.212)	(−40.754)	(6.352)	(0.954)
roa	15.534^***^	12.067^***^	15.545^***^	0.441^*^	1.111^***^
	(61.358)	(51.219)	(61.398)	(1.752)	(2.991)
size	0.099^***^	0.154^***^	0.103^***^	0.825^***^	0.435^***^
	(5.505)	(8.586)	(5.657)	(57.961)	(20.723)
cashflow	1.471^***^	−0.268^***^	1.470^***^	0.007	−0.141^*^
	(21.028)	(−2.864)	(21.017)	(0.121)	(−1.669)
growth	−0.006	−0.004	−0.006	−0.001	−0.009^**^
	(−1.570)	(−1.362)	(−1.606)	(−0.258)	(−2.179)
top1	0.005^***^	0.006^***^	0.005^***^	−0.001	0.001
	(4.570)	(5.462)	(4.580)	(−1.284)	(0.920)
soe	0.019	0.071^*^	0.022	0.099^***^	−0.288^***^
	(0.478)	(1.802)	(0.550)	(3.051)	(−6.057)
age	27.036^***^	23.627^***^	26.928^***^	23.750^***^	118.224^***^
	(4.019)	(3.746)	(4.005)	(4.458)	(15.015)
Ind FE	Y	Y	Y	Y	Y
Year FE	Y	Y	Y	Y	Y
Prov FE	Y	Y	Y	Y	Y
Adjust.R^2^	0.8553	0.7882	0.8553	0.5566	0.2768
F	2935.6249^***^	1281.5965^***^	2937.2774^***^	391.9669^***^	472.0568^***^
N	8,672	8,316	8,673	8,606	8,673

*t*-statistics in parentheses.

* *p* < 0.1;

** *p* < 0.05;

*** *p* < 0.01.

Furthermore, it needs to be proved empirically whether the risk caused by the high supplier concentration leads to the reduction of firm innovation. The study tests the relation between the risk and innovation measuring by *lnRD* and *allPatent*. The results are presented in columns (4)–(5) of Table 10. The coefficients of *Z-score* on *lnRD* and *AllPatent* are 0.053 and 0.019, it is positive and highly significant. The results suggest that the risk higher (that is, Z-score is lower), the R&D and patents are less. It implies that the supplier concentration leads to risk, further the risk reducing the innovation.

### Resource occupied mechanism

Based on the resource dependence theory (RDT) and the incomplete contracts theory, bargaining power imbalance exists between firms and its major suppliers as analyzed in the section II of “theoretical analysis.” The major supplier gains pricing power when a firm over depends on them, then they may occupy the firm’s resources through credit trade such as advance payment, impair the firm’s profit through transfer costs, thereby the firm does not have enough resources to invest R&D. So, the research predicts that it is a positive relation between firm’s supplier concentration and its prepayment. In order to verify the conjecture, the study use advance payment as the proxy variable of resource occupied. Especially, using the ratio of prepayment to total asset as is the explained variable (*YUF*), then regresses the supplier concentration and prepayment.

The results as shown in columns (1)–(3) of Table 11. As expected, the tests show a positive relation between supplier concentration measuring by dum1, SC1, and SC2 and resource occupied measuring by *YUF*. The coefficient of dum1, SC1, and SC2 on YUF are 0.003, 0.007, and 0.007. They are significantly positive, both economically and statistically. These findings suggest that a firm with higher supplier concentration, the firm’s resource occupied more by the major suppliers.

**Table 11:** Resource occupied mechanism.

	(1) YUF	(2) YUF	(3) YUF	(4) lnRD	(5) AllPatent
dum1	0.003^***^				
	(3.613)				
SC1		0.007^**^			
		(2.303)			
SC2			0.009^***^		
			(3.513)		
YUF				−0.092^**^	−1.894^***^
				(−2.232)	(−3.242)
lev	0.030^***^	0.023^***^	0.030^***^	−0.645^***^	−0.377^***^
	(11.320)	(7.900)	(11.308)	(−7.386)	(−2.906)
ldbl	−0.000	−0.000	−0.000	−0.009^***^	−0.025^***^
	(−0.514)	(−1.190)	(−0.712)	(−3.033)	(−5.878)
zbmjd	0.000^***^	0.000^***^	0.000^***^	0.001^***^	0.000
	(4.925)	(11.204)	(4.872)	(4.400)	(0.568)
roa	0.037^***^	0.018^***^	0.038^***^	1.212^***^	1.518^***^
	(6.405)	(2.911)	(6.445)	(6.348)	(5.352)
size	−0.000	0.001	0.000	0.834^***^	0.441^***^
	(−0.331)	(1.101)	(0.108)	(60.451)	(21.584)
cashflow	−0.002	−0.017^***^	−0.002	0.087	−0.126
	(−1.274)	(−6.768)	(−1.308)	(1.601)	(−1.552)
growth	0.000	0.000	0.000	−0.001	−0.009^**^
	(1.357)	(1.168)	(1.353)	(−0.404)	(−2.197)
top1	0.000^**^	0.000^***^	0.000^**^	−0.001	0.001
	(2.099)	(2.580)	(2.176)	(−1.088)	(0.903)
soe	−0.002^**^	−0.004^***^	−0.002^**^	0.087^***^	−0.324^***^
	(−2.448)	(−3.262)	(−2.276)	(2.779)	(−6.990)
age	0.019	−0.112	0.006	24.205^***^	116.779^***^
	(0.120)	(−0.644)	(0.036)	(4.665)	(15.154)
Ind FE	Y	Y	Y	Y	Y
Year FE	Y	Y	Y	Y	Y
Prov FE	Y	Y	Y	Y	Y
Adjust.R^2^	0.1499	0.1835	0.1498	0.5670	0.2767
F	735.1246^***^	682.1964^***^	915.2713^***^	413.8387^***^	676.9060^***^
N	7,024	5,512	7,025	6,957	7,025

*t*-statistics in parentheses.

* *p* < 0.1;

** *p* < 0.05;

*** *p* < 0.01.

Furthermore, the study examines the relation between the resource occupied by the major suppliers and firm’s innovation measuring by *lnRD* and *allPatent*. The research predicts that it is negative relationship between them. The columns (4)–(5) of Table 11 report the regression results. Consistent with the prediction, the coefficients of YUF on *lnRD* and *allPatent* are 0.092 and –1.894, both significantly positive in economically and statistically. It suggests that when a firm relies on major supplier, the firm may force to providing more prepayment, further occupying the firm’s resources, so the firm reduces resources on the innovation.

Overall, supplier concentration affects corporate innovation through risk and resource occupied mechanism. When supplier concentration is higher, the firm faced higher risk and be occupied more resource by the major suppliers, the above things further impact the firm’s R&D investment.

## Conclusion

Recent literature argues that a firm’s customer concentration impacts its financial decisions. However, they overlook how a firm’s supplier concentration reshapes the firm’s financial decisions. The purpose of this study is to explore how a firm’s supplier concentration affects its innovation. Using theoretical framework including resource dependence theory and theory of incomplete contracts, relying on the purchase ratio of top five suppliers and largest supplier as proxies for supplier concentration, the empirical investigation reveals that a firm with higher supplier concentration spends less on R&D investment and apply less patents. These findings imply that it is a risky for a firm over depending on their major suppliers.

Furthermore, the study discusses the mechanism that contributes to these findings. Namely, which mechanism drives the negative relation between supplier concentration and innovation. First, in line with the RDT, a firm over-relies on their major supplier, the firm faces high risk derive from the big suppliers. As a result, the firm cannot afford to take the risk of innovation. So, the firm reduces the R&D investment. Second, based on the theory of incomplete contract, when a firm over-relies on their major suppliers, these suppliers gain strong bargaining power, they will occupy the firm’s sources. Thereby, the firm does not have enough resources to invest innovation.

Due to the limitations of data availability and openness, there are still some defects in this paper. First, there may be some improvement in the measurement of supplier concentration in this paper. Second, this paper mainly focuses on empirical research and lacks mathematical deduction. The research in the future can enrich the measurement of supplier concentration, and expand the research on the influence and mechanism of supplier concentration on firms’ other financing decisions.

## Data availability statement

The original contributions presented in the study are included in the article/supplementary material, further inquiries can be directed to the corresponding author.

## Author contributions

MZ: acquisition of data and drafting the manuscript. XZ: revising the manuscript critically for important intellectual content and management and coordination responsibility for the research activity planning and execution. All authors contributed to the article and approved the submitted version.

## Funding

This research was funded by the general project of the National Natural Science Foundation of China (72171162), Taiyuan University of Science and Technology Scientific Research Initial Funding(TYSUT SRIF: 20222004), the Key Research Bases for Humanity and Social Science of Shanxi Province(20210132)and the Teaching Reform and Innovation Project of Taiyuan University of Science and Technology (XJ2021104).

## Conflict of interest

The authors declare that the research was conducted in the absence of any commercial or financial relationships that could be construed as a potential conflict of interest.

## Publisher’s note

All claims expressed in this article are solely those of the authors and do not necessarily represent those of their affiliated organizations, or those of the publisher, the editors and the reviewers. Any product that may be evaluated in this article, or claim that may be made by its manufacturer, is not guaranteed or endorsed by the publisher.

## References

[ref2] Acharya V. Xu Z. X. (2017). Financial dependence and innovation: the case of public versus private firms. J. Financ. Econ. 124, 223–243. doi: 10.1016/j.jfineco.2016.02.010

[ref3] Aggarwal V. A. Hsu D. H. (2014). Entrepreneurial exits and innovation. Manag. Sci. 60, 867–887. doi: 10.1287/mnsc.2013.1801

[ref4] Aghion P. Bloom N. Blundell R. Griffith R. Howitt P. (2005). Competition and innovation: an inverted-U relationship. Q. J. Econ. 120, 701–728. doi: 10.3386/w9269

[ref5] Aghion P. Van Reenen J. Zingales L. (2013). Innovation and institutional ownership. Am. Econ. Rev. 103, 277–304. doi: 10.1257/aer.103.1.277

[ref7] Altman E. I. (2005). An emerging market credit scoring system for corporate bonds. Emerg. Mark. Rev. 6, 311–323. doi: 10.1016/j.ememar.2005.09.007

[ref9] Autor D. Dorn D. Hanson G. H. Pisano G. Shu P. (2020). Foreign competition and domestic innovation: Evidence from US patents. Am. Econ. Rev. Insights 2, 357–374. doi: 10.1257/aeri.20180481

[ref10] Balsmeier B. Fleming L. Manso G. (2017). Independent boards and innovation. J. Financ. Econ. 123, 536–557. doi: 10.1016/j.jfineco.2016.12.005

[ref11] Banerjee S. Dasgupta S. Kim Y. (2008). Buyer-supplier relationships and the stakeholder theory of capital structure. J. Financ. 63, 2507–2552. doi: 10.2139/ssrn.607582

[ref12] Bloom N. Schankerman M. Reenen J. V. (2013). Identifying technological spillovers and product market rivalry. Econometrica 81, 1347–1393. doi: 10.3982/ECTA9466

[ref13] Bradley D. Kim I. Tian X. (2017). Do unions affect innovation? Manag. Sci. 63, 2251–2271. doi: 10.2139/ssrn.2232351

[ref14] Campello M. Gao J. (2017). Customer concentration and loan contract terms. J. Financ. Econ. 123, 108–136. doi: 10.1016/j.jfineco.2016.03.010

[ref16] Chemmanur T. Loutskina E. Tian X. (2014). Corporate venture capital, value creation, and innovation. Rev. Financ. Stud. 27, 2434–2473. doi: 10.1093/rfs/hhu033

[ref17] Chu Y. (2012). Optimal capital structure, bargaining, and the supplier market structure. J. Financ. Econ. 106, 411–426. doi: 10.1016/j.jfineco.2012.05.010

[ref18] Chu Y. Q. Tian X. Wang W. Y. (2019). Corporate innovation along the supply chain. Manag. Sci. 65, 2445–2466. doi: 10.1287/mnsc.2017.2924

[ref20] Custódio C. Ferreira M. A. Matos P. (2019). Do general managerial skills spur innovation? Manag. Sci. 65, 459–476. doi: 10.1287/mnsc.2017.2828

[ref22] Dhaliwal D. Judd J. S. Serfling M. Shaikh S. (2016). Customer concentration risk and the cost of equity capital. J. Accout. Econ. 61, 23–48. doi: 10.1016/j.jacceco.2015.03.005

[ref24] Fang L. H. Lerner J. Wu C. P. (2017). Intellectual property rights protection, ownership, and innovation: evidence from China. Rev. Financ. Stud. 30, 2446–2477. doi: 10.3386/w22685

[ref26] Fee C. E. Hadloc K. C. J. Thomas S. (2006). Corporate equity ownership and the governance of product market relationships. J. Financ. 61, 1217–1251. doi: 10.1111/j.1540-6261.2006.00871.x

[ref27] Ferreira D. Manso G. Silva A. C. (2014). Incentives to innovate and the decision to go public or private. Rev. Financ. Stud. 27, 256–300. doi: 10.1093/rfs/hhs070

[ref28] Grossman S. J. Hart O. D. (1986). The costs and benefits of ownership: a theory of vertical and lateral integration. J. Polit. Econ. 94, 691–719. doi: 10.1086/261404

[ref30] Hertzel M. G. Li Z. Officer M. S. Rodgers K. J. (2008). Inter-firm linkages and the wealth effects of financial distress along the supply chain. J. Financ. Econ. 87, 374–387. doi: 10.1016/j.jfineco.2007.01.005

[ref32] Huang H. H. Lobo G. J. Wang C. Xie H. (2016). Customer concentration and corporate tax avoidance. J. Bank. Financ. 72, 184–200. doi: 10.1016/j.jbankfin.2016.07.018

[ref34] Itzkowitz J. (2013). Customers and cash: how relationships affect suppliers' cash holdings. J. Corp. Financ. 19, 159–180. doi: 10.1016/j.jcorpfin.2012.10.005

[ref37] Kortum S. Lerner J. (2000). Assessing the contribution of venture capital to innovation. RAND J. Econ. 31, 674–692. doi: 10.7441/joc.2021.01.09

[ref38] Lerner J. (2009). The empirical impact of intellectual property rights on innovation: puzzles and clues. Am. Econ. Rev. 99, 343–348. doi: 10.2307/2696354

[ref39] Lian Y. (2017). Financial distress and customer-supplier relationships. J. Corp. Financ. 43, 397–406. doi: 10.1016/j.jcorpfin.2017.02.006

[ref40] Luong H. Moshirian F. Nguyen L. Tian X. Zhang B. (2017). How do foreign institutional investors enhance firm innovation? J. Financ. Quant. Anal. 52, 1449–1490. doi: 10.2139/ssrn.2409909

[ref43] Patatoukas P. N. (2011). Customer-base concentration: implications for firm performance and capital markets. Account. Rev. 87, 363–392. doi: 10.2308/accr-10198

[ref44] Pearson T. Trompeter G. (1994). Competition in the market for audit services: the effect of supplier concentration on audit fees. Contemp. Account. Res. 11, 115–135. doi: 10.1111/j.1911-3846.1994.tb00439.x

[ref45] Pfeffer J. Salancik G. (1978). The External Control of Organizations: A Resource Dependence Perspective. eds. J. Greenman, R. E. Beach New York, NY: Harper & Row.

[ref46] Tan J. S. Cao H. J. Kong X. J. (2019). Do major customers promote firms’ innovation? China J. Account. Res. 12, 209–229. doi: 10.1016/j.cjar.2019.01.003

[ref48] Tian X. Wang T. (2014). Tolerance for failure and corporate innovation. Rev. Financ. Stud. 27, 211–255. doi: 10.2139/ssrn.1399707

[ref50] Willekens M. Achmadi C. (2003). Pricing and supplier concentration in the private client segment of the audit market: market power or competition? Int. J. Account. 38, 431–455. doi: 10.1016/j.intacc.2003.09.002

[ref51] Williams H. L. (2013). Intellectual property rights and innovation: evidence from the human genome. J. Polit. Econ. 121, 1–27. doi: 10.1086/669706, PMID: 24639594 PMC3955392

[ref52] Zhang X. D. Zou M. F. Liu W. M. Zhang Y. F. (2020). Does a firm’s supplier concentration affect its cash holding? Econ. Model. 90, 527–535. doi: 10.1016/j.econmod.2020.01.025

